# Antimicrobial Susceptibility and Molecular Characterization of *Escherichia coli* Recovered from Milk and Related Samples

**DOI:** 10.3390/microorganisms10071335

**Published:** 2022-07-01

**Authors:** Frederick Adzitey, Saniyatu Yussif, Roland Ayamga, Sumaila Zuberu, Francis Addy, Gideon Adu-Bonsu, Nurul Huda, Rovina Kobun

**Affiliations:** 1 Department of Animal Science, University for Development Studies, Tamale P.O. Box TL 1882, Ghana; adzitey@yahoo.co.uk; 2 Department of Food Science and Technology, University for Development Studies, Tamale P.O. Box TL 1882, Ghana; saniyayussif@gmail.com; 3 Department of Veterinary Science, University for Development Studies, Tamale P.O. Box TL 1882, Ghana; rollenbozz@gmail.com (R.A.); zuberusumaila32@gmail.com (S.Z.); 4 Department of Biotechnology, University for Development Studies, Tamale P.O. Box TL 1882, Ghana; faddy@uds.edu.gh (F.A.); gideon.adb@gmail.com (G.A.-B.); 5 Faculty of Food Science and Nutrition, Universiti Malaysia Sabah, Jalan UMS, Kota Kinabalu 88400, Malaysia; rovinaruby@ums.edu.my

**Keywords:** antimicrobial, *E. coli*, Ghana, partial sequencing, milk, molecular

## Abstract

There is a rising concern about illnesses resulting from milk consumption due to contamination by pathogenic microorganisms including *Escherichia coli.* This study examined the occurrence and antimicrobial susceptibility of *E. coli* isolated from cow milk and related samples. Furthermore, partial sequencing was done to ascertain the genetic relatedness and possible cross contamination among the samples. In all, 250 samples, that is, 50 each of raw milk, cow teat, milkers’ hands, milking utensils, and fecal matter of cows, were cultured for the identification of *E. coli. E. coli* was detected in 101/250 samples (40.4%). Milk and fecal samples recorded the highest percentages of 68.0% and 66.0%, respectively. Forty-two (42) *E. coli* strains examined for antimicrobial resistance showed an overall 25.5% resistance, 15.0% intermediate resistance, and 59.5% susceptibility. The isolates had a high level of resistance to teicoplanin (100.0%), but were susceptible to chloramphenicol (95.2%) and azithromycin (92.9%). The Multiple Antibiotic Resistance (MAR) index pattern ranged from 0.1 to 0.5, and 40.5% exhibited multiple drug resistance. The *E. coli* strains formed 11 haplotypes, and a phylogenic tree analysis showed relatedness among the isolates in other African countries. This observation is an indication of cross contamination among the milk and its related samples.

## 1. Introduction

Milk and dairy products are consumed on a daily basis by billions of people all over the world because of its important nutritional components such as proteins, lipids, minerals, and vitamins that support the maintenance and growth of the body [1]. However, the consumption of milk comes with a risk as raw (unheated or unpasteurized) milk can contain pathogens which could be due to contamination from the animal or environment as a result of poor handling [2]. Soomro et al. [3] also indicated that the presence of pathogenic microorganism in milk has become a major public health problem, particularly among people who continue to consume raw milk. Among these microorganisms is pathogenic *E. coli* in inadequately pasteurized milk, which has been linked to foodborne outbreaks and the development of antimicrobial resistances [4,5].

Antimicrobial development and eventual clinical adoption is one of the most significant issues in medical history, with engineered medicines having saved millions of lives against diseases that would have been lethal [6]. Nonetheless, due to the development of multidrug resistance (MDR) in these pathogens, treating infectious diseases is becoming increasingly difficult. Between 1917 and 2017, humans’ understanding of the bacteria found in milk, as well as the techniques available to research into them, have drastically evolved [7]. Historically, a convectional or cultural method was used to isolate and to identify microorganisms such as *E. coli,* but this method is quite lengthy and does not identify microorganisms up to the strain level. Molecular methods, including gene amplification and sequencing, have enabled the identification of new pathogens as disease agents, allowing researchers to better classify microbes from cultures [8]. Furthermore, sequence analysis of conserved genes has been a reliable, accurate, inexpensive, and scalable method of microbial identification in environmental and health sciences over the last two decades [9].

Studies on the isolation, antimicrobial susceptibility, and sequencing of microorganisms from milk and related samples are available worldwide. Such studies in recent times were conducted by Gebeyehu et al. [10] in Africa, Hassani et al. [11] in Asia, Manishimwe et al. [12] in America, and Tóth et al. [13] in Europe. A one health approach to studying microorganisms, how they spread, their resistance behavior and how they relate genetically to others will contribute to reducing the incidence of their growing menace. Nonetheless, studies on the incidence of resistant *E. coli* in milk and related samples is limited in Ghana. This study was therefore carried out to determine the occurrence of *E. coli* recovered from milk and related samples. The study also determined the antimicrobial susceptibility and characterization of isolated *E. coli* using partial sequencing to ascertain their genetic diversity.

## 2. Materials and Methods

### 2.1. Study Area and Sample Collection

Raw cow milk and related samples were randomly collected from different locations in the Saboba district, Ghana from January to October, 2021. In all, two-hundred-and-fifty (250) samples composed of milk (*n* = 50), teat (*n* = 50), hands (*n* = 50), utensils (*n* = 50), and fecal matter (*n* = 50) were collected. The samples were kept in a cool box with ice packs and transported to the UDS Spanish Laboratory for analysis.

### 2.2. Isolation and Identification of E. coli

Milk (10 mL) samples were grown in 90 mL Buffered Peptone Water (BPW, Oxoid Limited, Basingstoke, UK) and incubated at 37 °C for 18–24 h. All other samples (swab samples) were grown at 37 °C for 18–24 h in 9 mL BPW. The subculture was streaked onto Eosin Methylene Blue (EMB) agar (Oxoid Limited, Basingstoke, UK) and incubated at 37 °C for 18–24 h [14]. On EMB agar, *E. coli* develops a strong acid that forms colonies with a green metallic sheen and a dark nucleated core. As a result, such isolates were sub-cultured on Nutrient agar (Oxoid Limited, Basingstoke, UK) for purification. They were initially confirmed using Gram stain and *E. coli* latex agglutination test (Oxoid Limited, Basingstoke, UK).

### 2.3. Antimicrobial Susceptibility of E. coli

The antibiotic susceptibility test was done using the disk diffusion method of Bauer et al. [15] after the confirmation of the isolates by PCR. The test was done to determine the antibiotic resistance of *E. coli* against the following antibiotics (classes); Ceftriaxone (Cro) 30 µg (Cephalosporins), Chloramphenicol (C) 30 µg (Chloramphenicol), Gentamicin (Gm) 10 µg (Aminoglycosides), Suphamethoxazole/trimethoprim (Sxt) 22 µg (Sulfonamides), Ciprofloxacin (Cip) 5 µg (Quinolones), Tetracycline (Te) 30 µg (Tetracyclines), Imipenem (Imi) 10 µg (Carbapenem), Amoxycillin (A) 30 μg (Penicillins), Azithromycin (Azm) 15 µg (Macrolides), and Teicoplanin (Tec) 30 µg (Glycopeptides). Purified cultures of *E. coli* were grown in Tryptic Soy Broth (TSB) (Oxoid Limited, Basingstoke, UK) at 37 °C overnight and the concentration was adjusted to 0.5 MacFarland turbidity. It was then spread plated on Muller Hinton Agar (MHA) (Oxoid Limited, Basingstoke, UK), and the antibiotic disks were placed on the surface of the inoculated plate at a distance to avoid the overlapping of inhibition zones. Plates were incubated at 37 °C for 24 h, and the results were interpreted according to the Clinical and Laboratory Standard Institute [16]. The Multiple Antibiotic Resistance (MAR) index was calculated and interpreted as a/b, where “a” represents the number of antibiotics to which the isolate was resistant, and “b” represents the number of antibiotics to which the isolate was exposed [17].

### 2.4. Molecular Identification

#### 2.4.1. DNA Isolation

Lysing was done by putting a colony of *E. coli* in 30 µL DNAse/RNAse free water and lysed at 99 °C for 30 min [18] in a thermocycler (peqSTAR 96X Universal gradient thermocycler, VWR, Darmstadt, Germany). The lysate was then used as the template for PCR amplification.

#### 2.4.2. PCR Amplification of Partial *uidA* Gene

Polymerase chain reaction (PCR) was done on the DNA. A partial fragment of 147 bp of the *uidA* gene was amplified using previously designed primers by Bej et al. [19], that is, *uidA*-F (5′-AAAACGGCAAGAAAAAGCAG-3′) and *uidA*-R (5′-ACGCGTGGTTAACAGTCTTGCG-3′). The PCR was performed in a total reaction volume of 50 µL, containing 10 µM each of forward and reverse primers, OneTaq^®^ Quick-Load 2x Master Mix with standard Buffer [20 mM Tris-HCl (pH 8.9 at 25 °C), 22 mM KCl, 1.8 mM MgCl_2_, 22 mM NH_4_Cl, 0.2 mM dNTPs, 5% glycerol, 0.05% Tween^®^ 20, 1.25 OneTaq^®^ DNA polymerase (New England Biolabs Inc., Ipswich, MA, USA)], and 5 µL of DNA. The PCR was performed under the following modified conditions [19]: initial denaturation at 94 °C for 5 min and then 95 °C for 30 s (denaturation); 57 °C for 30 s (annealing); and 72 °C for 30 s (extension) for 35 cycles, followed by a final extension of 72 °C for 5 min. A negative control (no DNA) was included to check for possible contamination in all reactions. The PCR amplicons were separated on 2% (*w*/*v*) agarose gel stained with ethidium bromide. The PCR products were finally visualized under UV light using UV Transilluminator.

#### 2.4.3. DNA Sequencing and Analysis

Twenty-four (24) PCR products were sequenced at Inqaba Biotechnology (Pty) Ltd. (Pretoria, South Africa). Gentle software v.1.9.4 http://gentle.magnusmanske, accessed on 8 January 2021 (Magnus Manske, University of Cologne, Köln, Germany) was used to view and clean DNA sequences and aligned using CLUSTAL W [20]. Identification was done by comparing individual sequences with previously deposited sequences in GenBank using the Basic Local Alignment Search Tool (BLAST) of the National Center for Biotechnology Information (NCBI). PopArt was used to construct a haplotype network of *uidA* gene sequences based on the TCS Algorithm [21], and DnaSP software [22] was used to determine the nucleotide and haplotype diversities of the sequences. The relationship of the sequence and sequences from other countries were shown with the Maximum Likelihood phylogenetic tree done by Molecular Evolutionary Genetics Analysis (MEGA X) software v.10.1 (Philadelphia, USA) [23].

### 2.5. Statistical Analysis

Data obtained from the isolation of *E. coli* was analyzed with the binary logistic of IBM Statistical Package for the Social Sciences (SPSS) Version 17 (New York, NY, USA). The test for statistical difference was done with wald chi-square at 5% significance level.

## 3. Results

### 3.1. Occurrence of E. coli in Milk and Related Samples

The occurrence of *E. coli* in raw milk (animal), the fecal matter of cows (animal), the utensils used for milking (environment), the teat of cows (animal), and the hands of the milking personnel (humans) is shown in Table 1. From a total of 250 samples taken from milk, feces, utensils, teat, utensils, and hands of milkers, 101 samples representing 40.4% tested positive for *E. coli.* The highest occurrence was recorded in milk with 34 positives representing 68.0%, followed by feces with 33 positives representing 66.0%. Furthermore, utensils recorded 21 positives representing 42.0%, followed by teat and hands with 7 (14.0%) and 6 (12.0%), respectively. Milk and fecal samples positive for *E. coli* were significantly higher (*p* < 0.05) than utensil, teat, and hand samples. Similarly, utensil samples positive for *E. coli* were significantly higher (*p* < 0.05) than teat and hands samples. Teat and hand samples did not differ significantly (*p* > 0.05) from each other.

### 3.2. Antimicrobial Susceptibility of E. coli Isolated from Raw Cow’s Milk and Related Samples

The antimicrobial susceptibility of the *E. coli* isolates is presented in Table 2. From Table 2, 25.5%, 15.0%, and 59.5% of the *E. coli* isolates were resistant, intermediate resistant, and susceptible, respectively. Out of the 42 isolates, 100.0% and 50.0% were resistant to teicoplanin and amoxycillin, respectively. The isolates were also susceptible to chloramphenicol (95.2%), azithromycin (92.9%), gentamycin (83.3%), imipenem (73.8%), sulphamethoxazole/trimethoprim (71.4%), tetracycline (61.9%), ceftriaxone (59.5%) and ciprofloxacin (54.8%).

### 3.3. Antimicrobial Resistance Profile and Multiple Antibiotic Index of Individual E. coli

The antibiotic resistance profile and multiple antibiotic resistant index of individual *E. coli* is shown in Table 3. From the table, seven (7) *E. coli* isolates were resistant to five (5) antibiotics, six (6) *E. coli* isolates were resistant to four (4) antibiotics, and four (4) *E. coli* isolates were resistant to three (3) antibiotics. Multidrug resistance occurs when a bacteria isolate exhibits resistance to three or more different classes of antibiotics. In this study, 17 (40.5%) *E. coli* isolates were resistant to three (3) or more different antibiotics.

### 3.4. PCR Amplification of uidA Gene for Confirmation of E. coli

**Figure 1:**
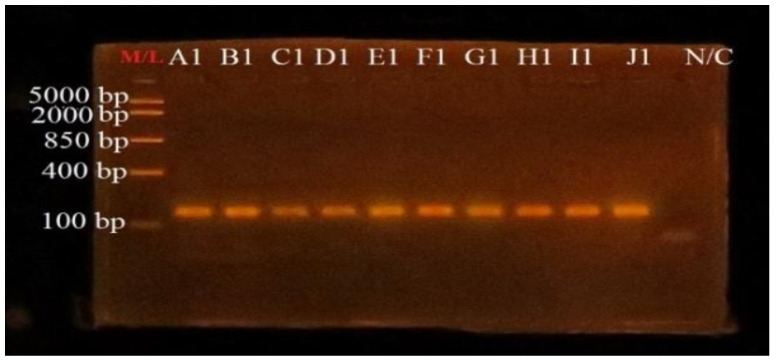
Agarose gel photo obtained from PCR products for identification of *E. coli*. M/L: Quick-Load^®^ Purple 100 bp DNA Ladder (New England Biolabs); A1 (Positive control, ATCC 25922); B1–J1 (*E. coli* isolates, ~147 bp); N/C: Negative control.

Polymerase chain reaction to confirm the *E. coli* isolates was performed using *uidA* specific primers to amplify partial fragment of the *uidA* gene. Agarose gel visualization showed successful amplification of ~147 bp fragment size as shown in Figure 1.

### 3.5. Sequencing and Species Identification

Out of the number of amplicons sent for sequencing, 22 samples were sequenced successfully and used for molecular analyses. All the 22 DNA sequences chromatogram obtained for molecular analyses were edited, and the sequences had a fragment length between 97 bp and 130 bp. All twenty-two sequences were queried through the NCBI BLAST algorithm for nucleotide comparison and species identification. It was confirmed that all isolates were *E. coli.* The sequences from the present study were 95.1–100% identical to already deposited sequences of *E. coli* in GenBank repository as shown in Table 4.

### 3.6. Haplotype Network Analysis and Indices

**Figure 2:**
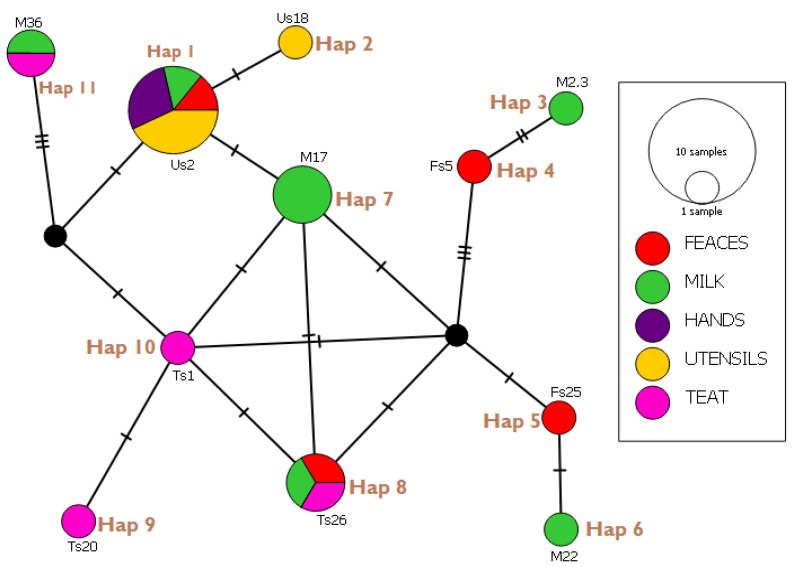
*E. coli uidA* (~147 bp) haplotype network of sequences from present study. Circle size indicate the frequency of haplotype in the dataset and the strokes refer to mutational steps.

All 22 sequences were subjected to haplotype network analysis to ascertain the indices and frequency of genes occurring between samples of the present studies. The results (Figure 2) indicated 11 haplotypes (hap01-11) with no singleton variable sites but nine (9) parsimony-informative sites/segregating sites. In all, 21 mutational steps were observed in the population indices with haplotype 1 and 3 showing the least and most mutational steps, respectively.

The overall indices of the genetic population studies of 22 *E. coli* samples isolated from milk and its related sources showed a haplotype diversity (Hd) of 0.877, nucleotide diversity (X) of 0.0360163, and variance of Hd as 0.00237. Haplotype 1 had the highest haplotype frequency of 31.8%, whereas haplotypes 2, 3, 4, 5, 6, 7, 8, 9 and 10 shared the least, with a haplotype frequency of 4.35%. 

### 3.7. Evolutionary Relationships (Phylogenetic Tree)

**Figure 3:**
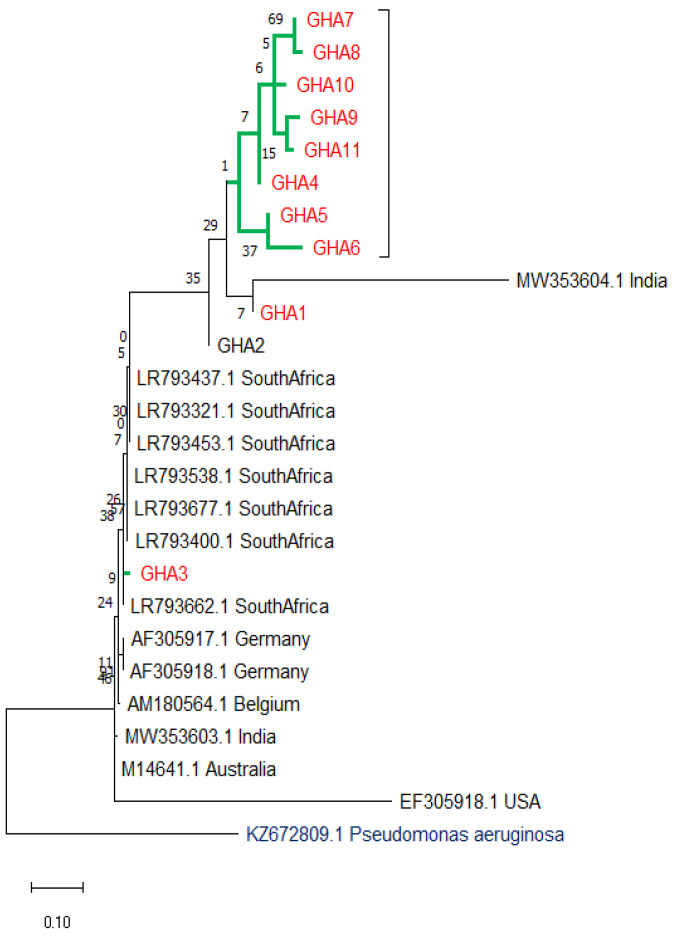
Maximum-likelihood Phylogenetic tree of *E. coli* based on the sequences of *uidA* gene sourced from repository of GenBank. Sequences of the present study are denoted GHA1-11 on the tree with *Pseudomonas aeruginosa* as outgroup.

The likelihood algorithm was used to infer the relationship between haplotypes of the present study and other studies around the globe as available in size and gene from the Gene Bank repository (Figure 3). Fourteen (14) *uidA* sequences from India, Australia, Belgium, Germany, and South Africa were included in the evolutionary analysis alongside 11 haplotypes (GHA 1–11) from the present study. Using the sequences of the present study, we noticed in Figure 3 that eight (GHA 4–11) clustered among themselves and remained at a distance from other repository sequences except MW353604.1 from India. However, GHA 7 and 8, GHA 9 and 11, and GHA 5 and 6 form monophyletic groups with themselves, respectively, as the clusters (GHA 4–11) formed a paraphyletic relationship. Those clusters shared a paraphyletic relation with GHA 1 and a polyphyletic relation with GHA 3. GHA 1 is seen to share a most common recent ancestor with MW353604, establishing them as monophyly. GHA 3 is seemingly quite related to sequences of other studies than those of the present study with evolutionary marker *uidA* in perspective. *Pseudomonas aeruginosa* (KZ672809) was used as outgroup. The tree shows 0.10 (10%) nucleotide substitution per site as indicated by the scale bar.

## 4. Discussion

In this study, cow milk and related sampes (i.e., cow feces, utensils for collecting milk, teat of the cows and hands of the cow milkers) were examined for the presence of *E. coli*. Overall, 40.4% of the samples were positive for *E. coli.* Ribeiro et al. [24] found *E. coli* in raw milk, feces, and water to be 74.6%, which was higher than that found in this study. The prevalence of *E. coli* was also higher in this study when compared with the 33.9% and 25.0% reported by Disassa et al. [25] and Yohannes [26], respectively. The results of this research were relatively similar to the 42.5% of *E. coli* reported for milk by Caine et al. [27]. It differs slightly from the reports by Samet Bali et al. [28], Yee et al. [29], and Salman and Hamad [30]; these studies reported lower incidences of *E. coli* in milk, with the percentages being 32.5%, 33.5% and 32.0%, respectively. In the present work, the highest occurrence was seen in milk with 68.0%, which corresponds to the figures recorded in a study by Fadaei [31], where *E. coli* was 69.0% in milk. Feces recorded the second highest with 66.0%, which is higher than the 21.2% reported by Beauvais et al. [32]. In this study, the high occurrence of *E. coli* in milk, fecal matter, and utensils is an indication that consuming raw milk could pose a threat to one’s life since there is a possibility of cross contamination from either of these sources. The high occurrence of *E. coli* in the utensils used for milking could stem from unhygienic practices [33].

Antimicrobial resistance is still a problem in the treatment of bacterial infections all over the world, especially where infections are common. Misuse/overuse of antibiotics by livestock farmers and poor surveillance systems leading to inadequate data have contributed to a rise in antimicrobial resistance rates in Ghana [34,35]. The risk factors linked with multidrug resistant (MDR) strains are also higher in developing nations than in industrialized countries [36]. Several risk factors associated with resistant *E. coli* colonization in cattle have been identified in previous studies investigating feed, milk, milking utensils, manure, flies, water, direct contact with infected animals, and animal wastes, all of which lead to the incidence and re-occurrence of *E. coli* infection and contamination of the animals and farm [37,38,39,40,41].

This study revealed a high resistance rate of *E. coli* to antibiotics such as teicoplanin and amoxycillin. However, they were susceptible to chloramphenicol, azithromycin, ciprofloxacin, gentamicin, imipenem, sulphamethoxazole/trimethoprim, tetracycline and ceftriaxone, which was similar to observations made by Adzitey [42], who also investigated samples from Ghana. Uddin et al. [43] isolated *E. coli* from raw milk in Dhaka, Bangladesh and reported that the isolates were 100.0% resistant against tetracycline, which was higher than that reported in this study. In addition, *E. coli* from milk samples were found to be resistant to amoxycillin and erythromycin [44]. Intermediate resistances were observed for amoxycillin, ceftriaxone, ciprofloxacin, gentamicin among others. Intermediate resistance refers to *E. coli* strains that are neither obviously resistant nor susceptible [42]. In clinical diagnosis, it has been proposed that patients with intermediate results should be given a larger dose of antibiotics [45]. Organisms with intermediate resistance are more likely to develop resistance quickly [46].

Multidrug resistance in *E. coli* strains has become a significant public health problem across the world in recent years. The multiple antibiotic (MAR) index varied from 0.1 (resistance to one antibiotic) to 0.5 (resistance to five antibiotics). Bacteria with a MAR index of greater than 0.2 comes from a high-risk source of frequent antimicrobial drugs usage or feed additives, whereas bacteria with a MAR index of less than 0.2 come from a source of infrequent antimicrobial drugs usage [47]. Multidrug resistance was 9.5%, 14.3%, and 16.7%, that is, resistance to three, four and five different antibiotics, respectively. Multidrug resistance is a source of worry since it restricts the therapeutic choices accessible for animals [48].

From 1986, *E. coli* has been an important determinant of human fecal contamination, as well as food- and water-related infections [49]. Universally, conventional and culture techniques with biochemical and serological tests are recognized as the gold standard methods for diagnoses and identification of *E. coli* [9,50]. However, this process is quite lengthy and may last 5–10 days or more and may not identify the microorganism to the serovar or strain level [51]. The commensality and versality of the pathogen makes it important for epidemiological and molecular pathogenic studies, especially when the pathogen’s genome is reported to be evolving constantly [50,52]. It is in this light that samples of isolated *E. coli* were subjected to genetic identification and characterization by amplification and analysis of the *uidA* gene, a unique genetic marker to *E. coli* [19,53]. Although haplotype studies done exclusively on the *uidA* marker for due comparison was not found, a low haplotype diversity was observed in the present study. This inference is made on a backdrop of 0.877 haplotype diversity. This is supported by 11 haplotypes forming out of 22 independent isolates with not more than 6 mutational steps between closer haplotype groups and 16 segregating sites. However, several population genetic studies in other study areas will be required for better appraisal of the claim. Interestingly, apart from Hap 07 (Figure 2), all haplotypes which were constituted by more than one isolate were from several sources of sample collection. For instance, members of Hap 01 are sourced from all five sources except from teat. Hap 08 has its members sourced from feces, milk, and teat. The case of Hap 01 with the highest haplotype frequency (31.8%) agrees to the assertion by Zhang et al. [54] that there can be cross infection from milkers or farmers to livestock and vice versa. However, several parameters can be included in future studies to validate the occurrence of sole infections of subjects and matters of such studies. It is worth noting that Hap 01 has the prevalent strain of *E. coli* in the study area. The case of Hap 8, 9, 10, 06, 05, 04, 03, and 11 may be attributed to the normal flora of the microorganism to the dairy cattle.

A phylogenetic tree constructed from partial *uidA* gene sequences from present GenBank showed that nine (9) out of the 11 haplotypes clustered among themselves. GHA7 and GHA8, GHA9 and GHA11, and GHA5 and GHA6, formed monophyletic relation, as all shared a common node. This is quite expected, as mutational sites among these sequences were ≤3. However, GHA1 formed a monophyletic relation with an Indian isolate (MW353604), indicating that they may share a recent common ancestry, which is possible by travel trials. But this and GHA3 positioning among sequences from South Africa, Germany, Belgium, India, and Australia cannot be readily explained especially with few DNA sequences employed. More data, better understanding of tradelines and livestock movement among these countries and Ghana, as well as in depth evolutionary studies are needed for better appraisal.

## 5. Conclusions

From the findings of this study, raw cow milk, cow fecal matter, cow teats, utensils for milking, and the hands of milkers were found to be contaminated with *E. coli.* Generally, more isolates were susceptible, followed by resistance and intermediate resistance. The isolates showed a high resistance to teicoplanin and amoxycillin. Furthermore, a higher susceptibility to imipenem, chloramphenicol, azithromycin, ciprofloxacin, gentamicin, sulphamethoxazole/trimethoprim, tetracycline, and ceftriaxone was observed. Relatively higher intermediate resistance to amoxycillin was observed. The haplotype networks indicate a cross contamination between the hands of the milkers, teat of the cow, feces, and the milking utensils. The phylogenetic tree shows how the Ghanaian isolates relate closely to themselves and those found in other African countries while differing from those found in Asian countries.

## Figures and Tables

**Table 1:** Occurrence of *E. coli* in raw cow’s milk and related samples.

Sample Type	Number of Samples	Number of Positives	% Occurrence
Milk	50	34	68.0
Feces	50	33	66.0
Utensils	50	21	42.0
Teat	50	7	14.0
Hands	50	6	12.0
Overall	250	101	40.4

**Table 2:** Percentage of Antimicrobial Susceptibility of *E. coli*.

Antibiotics	% Resistance	% Intermediate Resistance	% Susceptibility
Amoxycillin (A) 30 µg	50.0	47.6	2.4
Azithromycin (Azm) 15 µg	0.0	7.1	92.9
Ceftriaxone (Cro) 30 µg	9.5	31.0	59.5
Chloramphenicol (C) 30 µg	2.4	2.4	95.2
Ciprofloxacin (Cip) 5 µg	19.0	26.2	54.8
Gentamicin (Gm) 10 µg	2.4	14.3	83.3
Imipenem (Imi) 10 µg	9.5	16.7	73.8
Teicoplanin (Tec) 30 µg	100.0	0.0	0.0
Tetracycline (Te) 30 µg	35.7	2.4	61.9
Sulphamethoxazole/trimethoprim (Sxt) 22 µg	26.2	2.4	71.4
Overall	25.5	15.0	59.5

**Table 3:** Antibiotic Resistance Profile and Multiple Antibiotic Resistant Index of Individual *E. coli*.

Isolate Code	Sources	No. of Antibiotics	Antibiotics Resistance	MAR Index
FS17	Fecal	2	Tec-Te	0.2
FS22	Fecal	5	A-Tec-T-Cro-Sxt	0.5
FS25	Fecal	1	Tec	0.1
FS34	Fecal	4	Cip-Tec-Te-Sxt	0.4
FS48	Fecal	4	Cip-Tec-Te-Sxt	0.4
FS5	Fecal	2	Tec-Imi	0.2
FS50	Fecal	1	Tec	0.1
FS6	Fecal	1	Tec	0.1
FS8	Fecal	1	Tec	0.1
HS1	Hand	2	A-Tec	0.2
HS11	Hand	4	Cip-A-Tec-Te	0.4
HS12	Hand	3	A-Tec-Te	0.3
HS18	Hand	1	Tec	0.1
HS3	Hand	3	A-Tec-Te	0.3
HS9	Hand	1	Tec	0.1
M15	Milk	1	Tec	0.1
M17	Milk	1	Tec	0.1
M2	Milk	1	Tec	0.1
M25	Milk	2	A-Tec	0.2
M39	Milk	4	A-Tec-Cro-Imi	0.4
M45	Milk	2	A-Tec	0.2
M50	Milk	4	A-Tec-Te-Sxt	0.4
M51	Milk	1	Tec	0.1
M6	Milk	5	A-Tec-C-Cro-Sxt	0.5
M9	Milk	5	A-Tec-Te-Gm-Sxt	0.5
TS1	Teat	1	Tec	0.1
TS10	Teat	4	A-Tec-Te-Imi	0.4
TS20	Teat	2	A-Tec	0.2
TS26	Teat	5	Cip-A-Tec-Te-Sxt	0.5
TS27	Teat	1	Tec	0.1
TS36	Teat	2	Cip-Tec	0.2
TS45	Teat	3	Tec-Cro-Sxt	0.3
TS9	Teat	3	A-Tec-Imi	0.3
US18	Utensils	2	Tec-Te	0.2
US2	Utensils	2	A-Tec	0.2
US24	Utensils	5	Cip-A-Tec-Te-Sxt	0.5
US3	Utensils	5	Cip-A-Tec-Te-Sxt	0.5
US30	Utensils	5	Cip-A-Tec-Te-Sxt	0.5
US31	Utensils	2	A-Tec	0.2
US34	Utensils	2	A-Tec	0.2
US49	Utensils	1	Tec	0.1
US5	Utensils	1	Tec	0.1

Key: Ceftriaxone (Cro) 30 µg (Cephalosporins), Chloramphenicol (C) 30 µg (Chloramphenicol), Gentamicin (Gm) 10 µg (Aminoglycosides), Suphamethoxazole/trimethoprim (Sxt) 22 µg (Sulfonamides), Ciprofloxacin (Cip) 5 µg (Quinolones), Tetracycline (Te) 30 µg (Tetracyclines), Imipenem (Imi) 10 µg (Carbapenem), Amoxycillin (A) 30 μg (Penicillins), Azithromycin (Azm) 15 µg (Macrolides), and Teicoplanin (Tec) 30 µg (Glycopeptides). MAR index = a/b, where “a” represents the number of antibiotics to which the isolate was resistant, and “b” represents the number of antibiotics to which the isolate was exposed [17].

**Table 4:** Nucleotide identity of *E. coli* isolates in the present study compared with reference gene in the GenBank.

Haplotypes (Isolates)	*E. coli* Strain Identified	Gene Bank Reference	Country	Percentage Identity (%)
Hap 01 (Us2, Us30, Us34, Hs1, Hs18, Fs6, M15)	STEC2017-197 RHB07-C16 ECS C054 O100:H21 strain Res 13-lact	CP075663.1 CP055973.1 AP024112.1 CP062889.1	Switzerland USA Japan Canada	98.97 100 100 98.97
Hap 02 (Us18)	KCJ3K291 L3Cip3	CP054407.1 CP062211.1	USA New Zealand	98.95 98.95
Hap 03 (M23)	STW0522-31 H20 MING6	AP022409.1 CP069677.1	Japan Poland	98.92 98.92
Hap 04 (Fs5)	O176:H45 strain MIN9 chromosome	CP069682.1	Poland	97.92
Hap 05 (Fs25)	19-5 chromosome V14 beta-D-glucuronidase gene	CP047010.1 MW353604.1	China India	98.91 98.91
Hap 06 (M22)	EH10-18-47 0126:H45 MING 10	CP063499.1 CP069677.1	Laos Poland	100 100
Hap 07 (M17, M45, M46)	STEC2018-553 WS0115A 65ECOLEC	CP075665.5 CP035882.1 CP070914.1	Switzerland Egypt Singapore	100 100 100
Hap 08 (Ts26, Fs50, M51)	STEC- 183 chromosome 039:H21 strain Res13-lact-PEB08-01tcmA_3	CP0756971.1 CP062865.1 CP059835.1	Switzerland Canada China	98.95 100 98.2
Hap 09 (Ts 20)	SH9PTE6 EF7-18-51	CP073768.1 CP063487.1	China Laos	100 100
Hap 10 (Ts1)	EcPF20 CP070920.1	CP071441.1 CP070920.1	USA Singapore	98.25 100
Hap 11 (M36, Ts 36)	TW10722 RH-048-MS 179 chromosomes	CP035841.1 CP050206.1 CP062924.1	Guinea Bissau Bangladesh Turkey	96.84 95.1 98.5

## Data Availability

No new data were created or analyzed in this study.
